# Wnt Signaling Cascade in Dendritic Cells and Regulation of Anti-tumor Immunity

**DOI:** 10.3389/fimmu.2020.00122

**Published:** 2020-02-17

**Authors:** Amol Suryawanshi, Mohamed S. Hussein, Puttur D. Prasad, Santhakumar Manicassamy

**Affiliations:** ^1^Department of Pathobiology, College of Veterinary Medicine, Auburn University, Auburn, AL, United States; ^2^Georgia Cancer Center, Augusta University, Augusta, GA, United States; ^3^Department of Biochemistry and Molecular Biology, Medical College of Georgia, Augusta University, Augusta, GA, United States; ^4^Department of Medicine, Medical College of Georgia, Augusta University, Augusta, GA, United States

**Keywords:** Wnt, dendritic cells, beta-catenin (β-catenin), tumor microenvironment (TME), immunotherapy, anti-tumor immunity, immunotherapy

## Abstract

Dendritic cells (DCs) control the strength and quality of antigen-specific adaptive immune responses. This is critical for launching a robust immunity against invading pathogens while maintaining a state of tolerance to self-antigens. However, this also represents a fundamental barrier to anti-tumor immune responses and cancer immunotherapy. DCs in the tumor microenvironment (TME) play a key role in this process. The factors in the TME and signaling networks that program DCs to a regulatory state are not fully understood. Recent advances point to novel mechanisms by which the canonical Wnt signaling cascade in DCs regulates immune suppression, and the same pathway in tumors is associated with the evasion of anti-tumor immunity. Here, we review these recent advances in the context of the pleiotropic effects of the Wnts in shaping anti-tumor immune responses by modulating DC functions. In addition, we will discuss how Wnt/β-catenin pathway in DCs can be targeted for successful cancer immunotherapy.

## Introduction

Dendritic cells (DCs) control the strength and quality of the adaptive immune response (1, 2). This is critical for launching robust immunity against invading pathogens while maintaining a state of tolerance to self-antigen (3, 4). This dichotomy assumes a particular significance in tumor immune surveillance, as tumors actively suppress immune response through multiple mechanisms by creating tolerance to their own antigen (2, 4). This also represents a fundamental barrier to successful cancer immune therapy (2, 4). Accumulating evidence suggest that DCs play a fundamental role in driving immune suppression against tumor-associated antigens (TAAs) (5–7). We now know that there are multiple subpopulations of DCs that differentially regulate anti-tumor immune responses, and that these subsets display tremendous functional plasticity in response to instructive signals from the tumor microenvironment (TME) and tumor vaccines (5–7). Although there has been much progress in understanding DCs-driven immune suppression, signaling networks and transcription factors within DCs that regulate these responses are not fully understood. Thus, understanding the molecular mechanisms by which DCs fine tune the anti-tumor immunity will be useful in the rational design of therapies against various tumors. Emerging studies suggest a fundamental role for the Wnt signaling cascade in shaping the functions of immune cells in the TME, particularly DCs, in this process. In addition, recent studies have shown that tumor cell-intrinsic Wnt signaling plays a key role in the evasion of anti-tumor immunity in several human cancers. Here, we review these studies, highlight unanswered questions, and offer a conceptual framework for understanding the Wnt-signaling-mediated control of anti-tumor immunity.

## Wnts in the TME

The TME is a distinctive niche that contains not only malignant cells but also cells of the immune system, the tumor vasculature, lymphatics, fibroblasts, perivascular stromal cells, and extracellular matrix components (8). Wnts are secreted lipid-modified cysteine-rich glycoproteins, and the TME contains high levels of the Wnt family of ligands (Wnts) (9). In the TME, malignant cells, stromal cells, DCs, and macrophages secrete Wnts (10). In humans, there are at least 19 different Wnt proteins all within 350–400 amino acids in length and 10 different cognate Frizzled (Fzd) receptors (9). The composition of Wnt proteins varies depending on the types of tumors. For example, Wnt3a and Wnt5a are expressed at higher levels in melanoma, whereas Wnt1 is highly expressed in lung adenocarcinoma. Wnts can exert autocrine effects on tumor cells and paracrine effects on immune cells. Although there has been much progress in understanding the role of the Wnt pathway in tumor development, progression, and metastasis, the role of this pathway in regulating anti-tumor immunity is only beginning to become better defined in the past few years.

**Figure 1:**
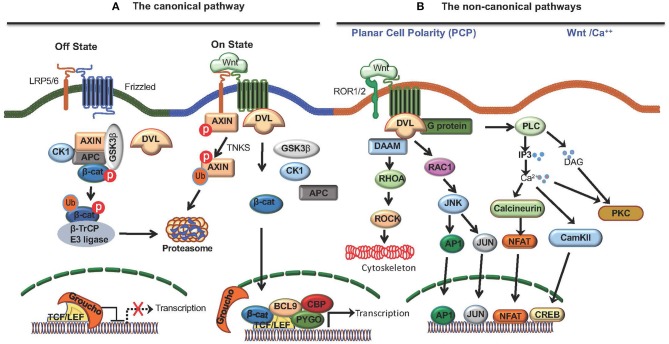
The Wnt signaling pathways. Wnt ligands bind to Frizzled (Fzd) receptors and activate the canonical Wnt pathway that is dependent on co-receptors LRP5/6 and β-catenin, and the non-canonical Wnt pathway that is independent of β-catenin. **(A)** The canonical Wnt pathway. In the absence of Wnt signaling (off state), free β-catenin in the cytoplasm is sequestered by adenomatus polyposis coli (APC)/Axin/CK1α/GSK3β complex, leading to its phosphorylation by glycogen synthase kinase 3β (GSK-3β) which targets it for degradation via the ubiquitin–proteosome pathway. Wnts binding Fzd receptors and LRP5/6 co-receptors (on state) recruits DVL resulting in disassembly of (APC)/Axin/CK1α/GSK3β complex, and the accumulation and translocation of unphosphorylated β-catenin to the nucleus. β-catenin translocation to nucleus results in the displacement of the co-repressor Groucho on the TCF/LEF transcription factor and the recruitment of co-activators, such as BCL9, CBP, and PYGO resulting in the transcription of target genes. Moreover, cytoplasmic TNKS ubiquitinates Axin, targeting it for proteasomal degradation and causing disassembly of the β-catenin destruction complex. **(B)** Wnts also activate non-canonical pathways, such as the planar cell polarity pathway and the Wnt-Ca^++^ pathway. The planar cell polarity pathway regulates cytoskeletal organization through Rhoa and Rock whereas the Wnt-Ca^++^ pathway activates several transcription factors, such as NFAT, AP1, JUN, CREB by β-catenin-independent mechanisms.

Wnt ligands bind to Fzd receptors and activate multiple signaling pathways that includes the canonical and non-canonical pathways (9). Co-receptors Low-density lipoprotein receptor-related protein 5 (LRP5) and LRP6 are critical for canonical Wnt signaling and β-catenin, a multifunctional protein, is a central component of this pathway (9). In the absence of Wnt-signaling, β-catenin is sequestered by adenomatus polyposis coli (APC)/Axin complex, leading to its phosphorylation by glycogen synthase kinase 3β (GSK-3β), which targets it for degradation via the ubiquitin–proteosome pathway (Figure 1). Wnt-signaling inactivates APC/Axin complex, resulting in the accumulation and translocation of unphosphorylated free β-catenin to the nucleus. In the nucleus, β-catenin interacts with T-cell factor/lymphoid enhancer factor (TCF/LEF) family of transcription factors and regulates transcription of several target genes (Figure 1) (9). In addition, Wnts can activate a number of non-canonical pathways, such as the planar cell polarity pathway and the Wnt-Ca^++^ pathways that activates several transcription factors, such as NFAT, AP1, JUN, CREB by β-catenin-independent mechanisms (Figure 1) (9). Recent reports show that tumor-intrinsic Wnt/β-catenin signaling facilitates immune evasion, whereas immune-cell intrinsic Wnt/β-catenin signaling drives immune suppression. However, the role of non-canonical Wnt signaling in regulating anti-tumor immune responses remains largely unexplored.

## DCs in the TME

DCs also play a fundamental role in maintaining the balance between immunity and tolerance (1, 3). DCs can be classified into distinct subsets, based on their phenotype, microenvironmental localizations, and functions. A detailed discussion of DC subsets and their influence on adaptive immunity is outside the scope of the present review, and the reader is referred elsewhere (5, 6). Briefly, in mice, multiple subsets of DCs exist in both lymphoid (CD8α^+^ DCs; pDCs; CD11b^+^ DCs) and non-lymphoid tissues (CD103^+^ DCs; CD11b^+^ DCs; pDCs). The TME contains all these major DC subsets. CD8α^+^/CD103^+^ are the migratory DCs that excel in transporting tumor-associated antigens (TAAs) to the lymph nodes in a CCR7-dependent manner (7, 11–13). They also display enhanced ability to prime and cross-present tumor antigens to CD8^+^ T cells. In general, CD11b^+^ DCs are more effective at driving CD4^+^ helper T cell responses; however, the role of these DCs in tumor immunity remain largely unexplored (14). The human counterparts of CD8α^+^/CD103^+^ and CD11b^+^ DCs have been identified, and they are CD141^+^ and CD1c^+^ DCs (14). Moreover, these DC subsets produce chemokines that are important for recruitment of CTLs to tumors (5, 7). Tumors also contain pDCs that are capable of producing high levels of type I interferons (IFN-I) in response to viral infection (5, 7, 15). Emerging studies have shown pDCs can drive effective anti-tumor specific immune responses where certain TLR ligands are used as tumor vaccine adjuvants (16). However, their role in anti-tumor immunity is still unclear and debatable.

## Modulation of DC Functions by Wnts Within Tumors

**Figure 2:**
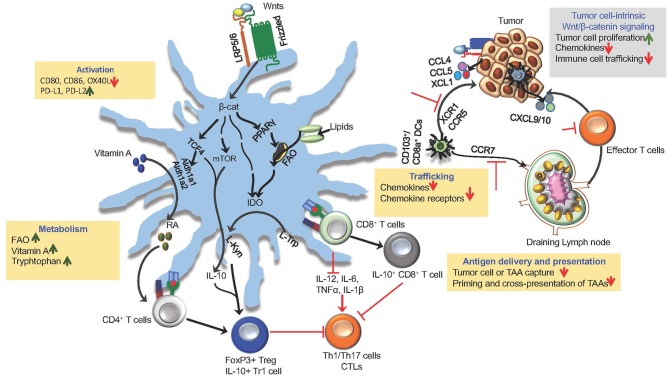
Wnt/β-catenin signaling in DCs and tumors in driving immune suppression and immune evasion. The canonical Wnt signaling shape anti-tumor immune responses by modulating DC functions, such as activation, trafficking, capturing and cross-presenting TAAs and expression of immune regulatory factors (RA, IL-10, IDO, TGF-β). Activation of β-catenin and its down-stream mediators mTOR and TCF4 in tumor DCs through Wnt-LRP5/6 signaling leads to the induction of anti-inflammatory factors RA, IDO, and IL-10 that are critical for promoting Treg response to tumor antigens. Wnts regulate trafficking of DCs to tumor and tumor-draining lymph nodes (TDLNs) by regulating the expression of chemokine receptors CCR5, XCR1, and CCR7. Within the TME, DC-intrinsic Wnt signaling limits the recruitment of CTLs by suppressing the expression of chemokines CXCL9/10. Wnt signaling leads to metabolic alterations in DCs that programs them to a regulatory state. Wnt-β-catenin-PPAR**γ**-mediated signaling shifts DC metabolism from glycolysis to FAO by upregulating the expression of the carnitine palmitoyltransferase-1A (CPT1A) fatty acid transporter. In addition, Wnt-β-catenin-TCF signaling in tumor DCs promote vitamin A metabolism through the induction of enzymes involved in RA synthesis and tryptophan metabolism through the induction of IDO. The canonical Wnt signaling pathway in tumor cells promotes tumor growth and immune evasion via chemokines (CCL4, XCL1, CCL5) that are critical for the recruitment and accumulation of DCs within the TME.

Robust anti-tumor immune response is dependent on several factors, such as the degree of maturation and activation of DCs, their ability to capture tumor cells and tumor-associated (TAAs), them trafficking to tumors and tumor draining lymph nodes (TDLNs), and type of factors they produce in the TME. However, DCs within the TME are often perceived as tolerogenic or immunosuppressive. In general, it is believed that instructive signals within the TME program DCs to a tolerogenic or immunosuppressive state rather than to an inflammatory state. A key issue is the nature of the instructive signals and molecules within the TME that promote regulatory DCs. Emerging studies have shown that canonical Wnt signaling plays a key role in shaping anti-tumor immune responses by modulating DC functions (Table 1; Figure 2). Here, we will discuss how Wnts shape key functions of DC in the TME that influence robust T cell- mediated cancer immunity.

**Table 1:** Evidence for involvement of the Wnt/β-catenin pathway in regulating immune suppression and immune cell exclusion.

**Observations**	**References**
**Wnt signaling regulating DC function**	
Wnt/β-catenin signaling regulates differentiation, maturation, and activation of DCs	(17–20)
Like tumor DCs, Wnt-conditioned DCs are programmed to a regulatory state to induce Tregs	(19)
Tumor DCs-deficient in LRP5/6 or β-catenin is more potent in capturing and cross-presenting TAAs to CD8^+^ T cells	(21–24)
Tumor DCs lacking LRP5/6 or β-catenin are programmed to induce Th1/Th17 cells	(21, 22)
Active Wnt/β-catenin signaling affects trafficking of DCs to tumors and TDLNs	(17)
Active Wnt/β-catenin signaling in tumor DCs regulates metabolic pathways involving FAO, vitamin A, and tryptophan to induce regulatory T cell (Treg) response	(21–26)
Wnt-signaling in tumor DCs suppresses chemokines that are critical of recruitment and accumulation of CTL in the TME	(17, 27, 28)
**Wnt signaling in T cells**	
Wnt/β-catenin signaling in Tregs promotes its survival, activity and infiltration	(29–31)
Wnt3a/β-catenin signaling suppresses effector T cell differentiation	(28, 32)
Wnt/β-catenin-signaling limits the expansion of tumor-antigen specific CD8^+^ T cells and is important in the maintenance of stemness of memory CD8^+^ T cells	(28, 32)
Wnt signaling in CD4^+^ T cells favors Th17 cell differentiation	(33, 34)
**Wnt signaling in macrophages**	
Wnt-β-catenin signaling regulates macrophages functions, such as adhesion, migration and tissue recruitment	(35, 36)
Wnt-b-catenin signaling promotes M2-like polarization of TAMs resulting in tumor growth, migration, metastasis, and immunosuppression	(35, 37, 38)
Wnts produced by macrophages drive contribute to tumor cell invasiveness and tumor growth	(35, 37, 38)
**Wnt signaling in MDSCs**	
The MUC1-β-catenin pathway regulates MDSC-mediated immune suppression in the TME	(39)
The PLCγ2-β-catenin pathway in MDSCs promotes tumor progression	(40, 41)
**Wnt signaling in NK cells**	
Wnt signaling in NK cells regulates maturation and effector functions	(42)
**Wnt signaling in tumor cells**	
Tumor growth, migration, and metastasis	(43–45)
Immune cell exclusion	(17, 27, 28, 46–48)

### Regulation of DC Maturation and Activation by Wnts

DC maturation and activation is an important step in inducing robust immune response against tumors. Immature or tolerogenic DCs facilitate tolerance toward tumors whereas immunogenic/inflammatory DCs facilitate robust anti-tumor responses (1, 49). Marked differences were observed in maturation and activation of status between tolerogenic/regulatory DCs and immunogenic/inflammatory DCs (1, 3). DCs in the TME have been shown to exhibit immature state or tolerogenic state (7, 49). Immature DC or regulatory DCs express markedly lower levels of costimulatory molecules (CD80, CD86, and CD40) whereas the immunogenic or inflammatory DCs express markedly higher levels costimulatory molecules (1, 3). DCs recognize a diverse array of microbial structures through multiple receptors collectively known as pattern recognition receptors (PRRs) (50). In addition, DCs can recognize damage-associated molecular patterns (DAMPs) and other endogenous ligands that are released from dying tumor cells through PPRs (51–53). PRRs include Toll-like receptors (TLRs), C-type lectin like receptors (CLRs), RIG-I like receptors (RLRs), and Nod-like receptors (NLRs) (50). TLR ligands have gained great interest in cancer immunotherapy in the recent years for their potential use as vaccine adjuvants (16, 54). PRR-mediated signaling controls DC functions, such as antigen uptake, antigen presentation, activation, and cytokine production that are critical for anti-tumor immunity (50). In general, PRR engagement potently activates DCs by upregulating the surface expression of maturation markers, such as MHCII, CD80, CD83, and CD86 (50). Even though, PRR ligands are there in the TME (51, 52), tumor-associated DCs display markedly decreased expression of co-stimulatory molecules (49). Emerging studies have shown an important role for Wnts in regulating maturation and activation of tumor-associated DCs. Earlier *ex vivo* studies on human and murine DCs have shown that exposure to Wnt1, Wnt3a, and Wnt5a that activates β-catenin can program DCs to a regulatory state with decreased expression of co-stimulatory molecules (17, 20, 21). Similar observations were made with murine and human DCs upon blocking GSK3β activation (a negative regulator of β-catenin) or activating β-catenin in DCs (55, 56). Such Wnt-conditioned regulatory DCs failed to upregulate co-stimulatory molecules even in response to TLR ligands (10, 57). Further, mechanistic studies have shown that the canonical Wnt signaling can negatively regulate the inflammatory pathways, such as the NF-kB and MAPK pathways, which are critical for DC activation (58). Accordingly, tumor DCs lacking LRP5/6 or β-catenin isolated from knockout mouse models displayed increased activation with upregulated expression of co-stimulatory molecule and decreased expression of co-inhibitory molecules (PD-L1, PD-L2) (21, 22). Furthermore, studies using small molecule inhibitors of canonical Wnt signaling in tumor bearing mice showed an augmented DCs activation with an increased expression of co-stimulatory molecules and decreased expression of co-inhibitory molecules (21, 22, 56). Collectively, these studies show that Wnt/β-catenin signaling interferes with DC maturation and activation in the TME.

### Regulation of DC Trafficking by Wnts

The migration of DCs is essential for tumor immune surveillance (5, 6). This involves, DCs migrating to tumor tissues, capturing and endocytosing dead tumor cells or cellular debris, and transporting TAAs to TDLNs where they prime and activate tumor-specific T cells (7, 11–13). This is dependent on the expression of specific chemokine receptors on DCs and its cognate chemokine ligand expression within the TME and TDLNs. The migration of DCs to TDLNs requires CCR7 expression whereas the recruitment of DCs to the TME is dependent on chemokines, such as CCL4, CCL5, and XCL1 (5). However, only a small fraction of DCs end up migrating to tumor tissue and subsequently to TDLNs. This is due to factors in the TME that control the expression of chemokine receptors and chemokines. Recent studies have shown that Wnts in the TME regulate DC trafficking by regulating chemokine receptors and chemokines via two different mechanisms. First, DC-intrinsic Wnt-signaling regulates its migration to tumors and TDLNs. Evidence supporting this come from studies showing that conditional deletion of either LRP5/6 or β-catenin in DCs in mice lead to marked increase in the number of DCs in TDLNs and TME. Furthermore, similar observations were made upon treating tumor-bearing mice with pharmacological inhibitors of the Wnt-β-catenin pathway (21, 22). Tumor DCs also produce chemokines that are critical for accumulation of T cells within the TME (5). A recent study on lung adenocarcinoma (LUND) has shown that tumor DC-intrinsic Wnt signaling plays a key role in blocking T cell infiltration into the tumors and driving cross-tolerance to tumor antigens (17). Mechanistically, Wnt1-mediated β-catenin signaling in tumor DCs resulted in transcriptional silencing of CC/CXC chemokines that are critical for recruiting effector T cells to the TME (17). In line with these observations, other studies have shown that the pharmacological blocking of the canonical Wnt signaling in DCs result in increased accumulation of effector T cells within the TME (21, 22). Second, tumor-intrinsic Wnt-signaling regulates the evasion of anti-tumor immunity by regulating the expression of chemokines that are critical for recruitment and accumulation of DCs in the TME (10). In this context, it was shown that an active Wnt-β-catenin signaling in melanoma cells suppresses production of CCL4 (C-C motif chemokine ligand 4) and consequently reduces migration and accumulation of CD103^+^ DCs in the TME (46). CD103^+^ DCs play an important role in recruitment of CTLs in TME through their production of CXCL9 (C-X-C motif chemokine ligand 9) and CXCL10 (46, 47). Collectively, these studies suggest that Wnt/β-catenin signaling regulates trafficking of DCs and T cells to the tumor tissue by regulating the expression of chemokine receptors and chemokines.

### Regulation of Antigen Delivery and Presentation by Wnts

Effective cross-priming of tumor-specific CD8^+^ T cells by DCs involves efficient capture and cross-presentation of tumor-associated antigens (2, 4, 5). However, DCs within the TME are less efficient in cross-priming CD8^+^ T cells (2, 4, 5). In addition to regulating DC trafficking, recent studies with fluorescently labeled proteins have shown that in the absence of the canonical Wnt signaling, DCs are more efficient in capturing TAAs and actively transporting them to the TDLNs (21, 22). These findings are further supported by observations that LRP5/6- or β-catenin- deficient tumor DCs are more potent in capturing TAAs and are robust in priming and cross-presenting TAAs to CD8^+^ T cells (23–25, 59). Similar observations were made upon pharmacologically blocking canonical Wnt signaling in tumor DCs (17, 23–25, 27, 48, 59). In contrast, Wnt-conditioned DCs or tumor DCs expressing a constitutively active form of β-catenin is less potent in capturing and cross-presenting TAAs (17, 21, 22, 27, 48). Collectively, these studies show that the activation of canonical Wnt signaling pathway in tumor DCs suppresses efficient capture of tumor-associated antigens and cross-priming of CD8^+^ T cells.

### Metabolic Reprogramming of DCs by Wnts

Aberrant Wnt signaling leads to metabolic alterations in cancer cells that are critical for their survival and proliferation (60). Interestingly, Wnt signaling also plays an important role in the metabolic reprogramming of DCs in the TME (60, 61). Cellular metabolic pathways play a critical role in modulating the functions of DCs (61–63). Emerging evidence show that potential metabolic differences exist among DC subsets and also between tolerogenic and immunogenic DCs (60, 62). Immature or tolerogenic DCs show a catabolic metabolism that is manifested by increased oxidative phosphorylation, fatty acid oxidation (FAO), and glutaminolysis (61, 63, 64). In contrast, immunogenic or inflammatory DCs display an anabolic metabolism that is marked by increased glycolysis (61, 63, 64). Accumulating evidence show that tolerogenic response to tumors is also related to the metabolic dysfunction and metabolic reprograming of immune cells within the TME (26, 61, 63, 64). A recent study has shown that Wnt-mediated signaling shifts DC metabolism from glycolysis to FAO by upregulating the expression of the carnitine palmitoyltransferase-1A (CPT1A), an enzyme important for the transport of fatty acids into mitochondria (26). Furthermore, this study also revealed that this metabolic shift is dependent on the activation of β-catenin and PPAR-γ in DCs (26). In addition, other studies have shown that the canonical Wnt signaling in tumor DCs promote the metabolism of vitamin A and tryptophan through the induction of enzymes involved in retinoic acid (RA) synthesis and indoleamine 2,3-dioxygenase-1 (IDO) (22, 25). Collectively, these studies have conclusively demonstrated that Wnt-mediated metabolic shift is critical for programming DCs to the regulatory state in the TME.

### Induction of Immune Regulatory Factors by Wnts

The types of cytokines and other factors secreted by DCs program the differentiation of newly primed CD4^+^ and CD8^+^ T cells into effector T cell or regulatory T cells (1, 3). The TME contains higher levels of immune regulatory factors, such as IL-10, RA, and TGF-β that actively suppress differentiation and expansion of tumor-specific effector T cells (10, 57). Given the enormous burden of endogenous PRR ligands, DCs associated with the tumors express higher levels of immune regulatory factors that drive Treg response (10, 57). Recent studies have highlighted an important role for the Wnt signaling in tumor-associated DCs in regulating the expression of immune regulatory factors (21–26). Unlike DCs stimulated with microbial products, Wnt-conditioned DCs do not release immunostimulatory cytokines; instead they express IDO, IL-10, RA synthesizing enzymes and TGF-β1 (21–26). In contrast, tumor DCs that are deficient in LRP5/6 or β-catenin expressed markedly higher levels of IL-12, IL-6, IL-23, and TNF-α, and lower levels of immune regulatory factors (21, 22). These observations where further corroborated by studies showing that pharmacological blocking of the Wnt/β-catenin pathway in tumor DCs decreased the expression of RA synthesizing enzymes, IDO, IL-10, and TGF-β1 while markedly increased the expression of inflammatory cytokines (21–26).

## Wnt Signaling Networks in DCs That Drive Treg Responses

The TME conditions DCs to acquire tolerogenic or immunosuppressive properties by activating the immune regulatory pathways (1, 5, 49). Although there has been much progress in understanding the role of DCs in inducing regulatory responses to tumor antigens, we understand very little about the regulatory signaling networks that program tumor DCs to become tolerogenic or immunosuppressive. In this context, there are emerging insights into the roles of the Wnt signaling network in DCs orchestrating tolerogenic responses to tumors.

### The Wnt-β-Catenin-RA Signaling Axis

First, a key mechanism by which Wnts in the TME drives immune suppression is through induction of vitamin A-metabolizing enzymes in DC via the β-catenin/TCF pathway (22). TME contains high levels of RA, and APCs are major producers of RA (22, 65). It is well-established that RA, an active metabolite of vitamin A, regulates a broad array of immune responses (66, 67). RA synthesis is a tightly regulated process that includes several key enzymes. Within cells, RA is produced from vitamin A (retinol) via a two-step enzymatic pathway where retinol is first oxidized to retinaldehyde (retinal) by alcohol dehydrogenases (ADH-1, -4, -5), which is next converted to RA by retinal dehydrogenases (Aldh1a1 and Aldh1a2) (50). In contrast to the gut DCs, DCs in the periphery do not express Aldh1a1 and Aldh1a2, but do constitutively express different isoforms of ADH, and hence, they lack the ability to convert vitamin A to RA (68, 69). However, within the TME, DCs express enzymes Aldh1a1 and Aldh1a2 and acquire the ability to metabolize Vitamin A to RA (22). RA produced by tumor DCs acts directly on CD4^+^ T cell cells, inducing Treg cell response while suppressing T effector cell response. Pharmacological blocking of RA synthesis or RA signaling affected the ability of tumor DCs to induce Treg (22). In line with these observations, DCs isolated form LRP5/6- or β-catenin- conditional knockout mice bearing B16F10, LLC, or EL4 tumors expressed markedly lower levels of vitamin A-metabolizing enzymes, and these DCs were less potent in inducing Tregs in response to tumor antigens (21, 22). Collectively these studies have shown that tumors, through DCs, exploit the LRP5/6-β-catenin-RA pathway as a mechanism of immune suppression by inducing regulatory T-cell responses.

### The Wnt-β-Catenin-PPARγ-IDO Signaling Axis

A second key mechanism by which Wnt-β-catenin signaling in DCs promotes immune suppression is through the induction of IDO (25, 26). IDO is an immunoregulatory enzyme that catalyzes the degradation of the essential amino acid tryptophan into kynurenines (59). Previous work has identified that tumor DCs express IDO and depletion of tryptophan dampens T-cell proliferation and the generation of kynurenine drives Treg differentiation (59). However, the signaling pathways in DCs that control IDO expression and its activity remained unknown. In this context, recent studies have shown an important role for the Wnt5a-β-catenin-PPARγ signaling pathway in regulating IDO expression and activity (25, 26). Furthermore, abrogation or blocking this pathway in a transgenic murine melanoma model markedly reduced IDO expression and activity in DCs with augmented anti-tumor immune responses (25, 26). These data indicate that Wnt5a-conditioned DCs promote the differentiation of Tregs in an IDO-dependent manner and that this process serves to suppress melanoma immune surveillance. Since Wnt5a activates the non-canonical Wnt pathway in DCs (18, 19), further mechanistic studies are necessary to understand how Wnt5a activates β-catenin in DCs within the TME. Also, additional studies are warranted to understand whether β-catenin directly regulates IDO expression in DCs through TCF4 or PPAR**γ**.

### The Wnt-β-Catenin-mTOR-IL-10 Signaling Axis

Finally, the Wnt-β-catenin pathway in DCs can drive T cell tolerance to tumors through the induction of IL-10. IL-10 is a key immunosuppressive cytokine that regulates a broad array of immune responses (70). Recent studies have shown that Wnt-β-catenin-dependent activation of mTOR and TCF4 in DCs regulates IL-10 expression (21, 23, 24). IL-10 produced by DCs exert autocrine effects to suppress cross-priming of tumor-specific CD8^+^ T cells (23). Furthermore, tumor DCs that are deficient in LRP5/6 or β-catenin express markedly lower levels of IL-10 and are more potent in cross-priming CD8^+^ T cells (21, 23, 24). In line with these observations, selective blocking of LRP5/6, β-catenin, and mTOR activation resulted in markedly reduced IL-10 production by DCs with augmented anti-tumor immune response in mice (21, 23, 24). However, further studies are warranted to understand mechanistically how β-catenin regulates mTOR activation in tumor DCs to induce IL-10. Collectively, these studies illustrate a critical role for the canonical Wnt signaling network in programming tumor DCs to induce regulatory response through induction of immune regulatory factors.

## Effects of Wnts in Modulating Functions of Other Immune Cells Within the TME

The role of the Wnt signaling cascade in other immune cells in promoting tolerogenic response is poorly understood. Beyond the ability to modulate DC functions, Wnts in the TME can directly influence the development and effector functions of various immune cell types. Wnt-mediated immunological tolerance to tumors is often considered to occur by multiple processes. Emerging studies are beginning to provide insights into the mechanisms by which Wnt signaling cascade directly modulates immunological functions of other immune cells, such as macrophages, myeloid-derived suppressor cells (MDSCs), Tregs, CD4^+^ T cells, CD8^+^ T cells, and NK cells (71). There are several excellent studies and reviews that discuss extensively how the Wnt signaling pathway shape the functions of other immune cells (43, 44, 60, 71, 72) and will thus be discussed only briefly (Table 1). Tumor-infiltrating T cells express markedly higher levels of Wnt3a and β-catenin, and display dysfunctional and exhausted effector memory phenotype (28). Furthermore, Wnt-mediated activation of β-catenin/TCF1 pathway activation suppresses naïve T cell differentiation and terminal effector differentiation of CD8^+^ T cells (28, 32). Likewise, T cell-infiltrating human hepatocellular carcinoma and colorectal cancer are dysfunctional and show an exhausted effector memory phenotype (43, 44, 60). In line with these observations, Wnt3a neutralization in tumor-bearing mice controls tumor growth by augmenting the expansion of tumor-antigen-specific CD8^+^ T cells with enhanced effector functions (27). Furthermore, the induction of Wnt signaling is critical for maintaining stemness of memory CD8^+^ T cells and Treg-intrinsic β-catenin signaling is critical for Treg survival, migration and suppressive functions (29–31). IL-17A-producing CD4^+^ T (Th17) cells play an important role in the pathogenesis of colorectal cancer (33, 34). In murine colorectal cancer model, forced expression of β-catenin in CD4^+^ T cells caused increased IL-17A expression that favor tumor progression. Natural killer T (NKT) cells are specialized CD1d-restricted T cells that play a critical role in tumor immune surveillance (73). Cytokines produced by activated NKT cells regulate the functions of other immune cells in the TME. There is ample evidence that Wnts can modulate anti-tumor immune response through NKT cells by suppressing the expression IFN**γ** (42). In addition, the chemokines CCL5 and XCL1 produced by NK cells also contribute to the recruitment and accumulation of DCs, macrophages, and Tregs within the TME (74, 75). However, it is not known whether Wnt signaling in NK cells regulates the expression of these chemokines. Tumor-associated macrophages (TAMs) play an important role in tumor progression and immune suppression (76–78). Wnts regulate macrophage functions, such as adhesion, migration, and tissue recruitment (35). In addition, it is well-documented that Wnts produced by macrophages is critical for tissue development and repair (35). Emerging studies have shown that Wnts produced by macrophages contribute to tumor cell invasiveness and tumor growth (36, 37). Furthermore, active Wnt-β-catenin signaling in macrophages programs to M2-phenotype that drives cancer cell growth, migration, metastasis, and immunosuppression (37, 38). MDSCs are a heterogeneous mix of cells that expand during cancer and potently suppress T cell responses (79, 80). Interestingly, in the murine extraskeletal tumor model, the PLCγ2-β-catenin pathway plays an important role in tumor progression, suggesting an anti-tumorigenic role for this pathway in MDSCs (40, 41). Conversely, in EL4 tumor model, the MUC1-β-catenin pathway is critical for MDSC development, suggesting a key role in MDSC-mediated immune suppression instead of tumor progression (39). Collectively, these studies show that in addition to DCs, Wnts in the TME can modulate anti-tumor immunity by directly regulating the effector functions of other immune cells.

## Targeting the Canonical Wnt Signaling Pathway for Cancer Immunotherapy

**Figure 3:**
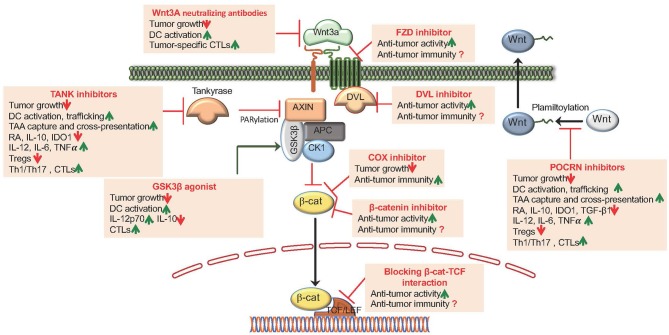
Pharmacological targeting of Wnt/β-catenin signaling augments anti-tumor activity and anti-tumor immune responses. The canonical Wnt signaling pathways can be targeted at four different levels: (1) Blocking Wnt ligand interaction with Fzd receptors by utilizing Wnt ligand antagonists that includes mAbs generated against the Wnts and Fzds and DKK1; (2) Blocking Fzd-LRP5/6 signaling by utilizing POCRN or DVL inhibitors; (3) Promoting β-catenin degradation by utilizing TNKS inhibitors, β-catenin-destruction complex activators that targets the Ser/Thr kinases CK1α or GSK3β and COX inhibitors and (4) Antagonizing β-catenin interaction with TCFs utilizing several small molecule modulators and synthetic inhibitors.

Accumulating evidence from studies involving human cancers suggest that enhanced Wnt signaling is associated with worst clinical outcomes. Hence, targeting the canonical Wnt signaling pathway is a promising approach to overcome immune evasion by tumors and to augment anti-tumor immunity by potently activating DCs. Pre-clinical studies have shown that the canonical Wnt signaling pathways can be targeted at four different levels to overcome tumor-mediated immune suppression and augment anti-tumor immunity. These strategies include (1) blocking ligand-receptor interaction, (2) blocking Fzd-LRP5/6 signaling (PORCN inhibitors), (3) promoting β-catenin degradation (tankyrase enzyme or TNKS inhibitors), and (4) blocking β-catenin-TCF interaction (β-catenin inhibitors) (Figure 3). Pharmacological inhibitors of the Wnt pathway exist and several of them are currently in clinical testing [extensively reviewed in (43–45)]. Here, we will briefly discuss pre-clinical studies related to effects of blocking the canonical Wnt pathway on anti-tumor immunity.

### Blocking Wnt Ligand Interaction With Fzd Receptors

Emerging studies indicate a strong correlation between specific Wnt ligand expression based on the type of tumor (43–45). Thus, blocking specific Wnt ligand interaction with cognate Fzd receptors represents a potential strategy to restrain tumor cell proliferation while boosting the anti-tumor immunity (43–45). In this context, a previous study (81) using a murine model of prostate cancer has shown that administration of Wnt3a-neutralizing antibodies restrained tumor growth. However, the impact on anti-tumor immunity was not assessed in this study (81). In another critical study, it was shown that administration of Wnt3a-neutralizing antibodies in tumor bearing mice restrains tumor growth by potently activating DCs and boosting the expansion of tumor-specific CD8^+^ T cells with improved effector activities (27). Likewise, neutralizing or silencing Wnt1 in lung adenocarcinoma model augmented DC activation, resulting in increased recruitment and accumulation of CTLs in the TME (17). In addition, In addition, monoclonal antibodies (mAbs) that targets the different Fzd receptors are in preclinical stage and early clinical trails in humans (43–45). There are also several recently developed therapeutic agents targeting dickkopf family members (DKK1) that inhibit the binding of Wnts to co-receptors LRP5/6 (43–45). However, further studies are warranted to understand the immune consequence of DKK1 as therapeutic agents in tumor settings.

### Blocking Fzd-LRP5/6 Signaling

Using clinically relevant murine tumor models, studies have examined anti-tumor immune responses by blocking Fzd-LRP5/6 signaling. Porcupine (POCRN) is a membrane-bound-O-acetyltransferase enzyme that palmitoylates Wnts, which is critical for its interactions with co-receptors LRP5/6 and Fzd receptors (10). Treatment of mice with established B16-OVA tumors or EL4-OVA tumors with IWP-L6 or C59 delayed tumor growth, and this was due to marked increase in tumor-antigen-specific CD4^+^ and CD8^+^ effector T cells with reduced number of Tregs, IL-10^+^ Tr1, and IL-10^+^ CD8 T cells within the tumors (21). Furthermore, IWP-L6 or C59 treatment enhances the ability of DCs to capture and cross-present tumor antigens to CD8^+^ T cells (71). Similar effect was observed in murine models of melanoma, colorectal, and ovarian cancer using different POCRN inhibitors, RXC004 and WNT974 (82). In addition, POCRN inhibitor in combination with checkpoint inhibitors, such as anti-PD-1, or chemotherapy was also found to enhance anti-tumor immunity (82).

### Promoting β-Catenin Degradation

Third strategy to augment anti-tumor immune responses involves promoting β-catenin degradation using TNKS inhibitors (43–45). TNKS enzymes are members of PARP family that regulate the canonical Wnt signaling via PARylation of AXIN, a key component of β-catenin destruction complex. TNKS inhibitors promote β-catenin degradation by increasing the levels of AXIN (43–45). Recent studies have shown that treatment of melanoma or EG7 tumor-bearing mice with TNKS inhibitors XAV939 or JW55 markedly delayed tumor growth with augmented anti-tumor immunity (21, 22, 24). Likewise, recent *ex vivo* study on co-culture of LNCaP and PC-3 prostate cancer, cells with lymphocytes from prostrate cancer patients have shown that lymphocytes treated with XAV 939 are more potent in eliminating LNCaP and PC-3 prostate cancer cells (83). In addition, XAV 939 in combination with vaccines enhances anti-tumor immune responses by potently activating DCs in mice (83). Similarly, RNAi-mediated inhibition of β-catenin resulted in marked increase in anti-tumor immune response with reduced tumor burden in models of B16F10 melanoma, 4T1 mammary carcinoma, Neuro2A neuroblastoma, and renal adenocarcinoma (48). Another potential approach to promote β-catenin degradation is by activating GSK3β (55). In this context, a recent study using murine melanoma model has shown that intratumoral activation of GSK3β improved tumor immune surveillance that is associated with increased DC activation and CD8^+^ effector response (55).

### Blocking β-Catenin-TCF Interaction

Finally, another potential strategy to target the canonical Wnt pathway is blocking the interaction of β-catenin with its downstream transcription factors (43–45). Several small molecule modulators and synthetic inhibitors that antagonize β-catenin interaction with TCFs have been identified and tested on murine tumor models of myeloma, liver, colorectal, and breast cancer (43–45). These studies have shown that blocking the interaction of β-catenin and TCF interaction markedly reduced the tumor growth (43–45). However, the primary focuses of these studies are mostly to test the effect on cancer stem cells and tumor cells, but not on immune cells. In this context, a recent study has shown the peptide-mediated targeted blocking of β-catenin interaction with BCL9 co-factor and its downstream transcription factor shows robust anti-tumor efficacy across multiple murine tumor models (56, 84). Markedly, this treatment approach augmented intratumoral infiltration of cytotoxic T cells by reducing Tregs and increasing DCs and also sensitizing cancer cells to PD-1 inhibitors (84). Further preclinical studies are warranted to understand the effect of other pharmacological inhibitors that targets β-catenin and TCF interaction on anti-tumor immunity.

Thus, pharmacological blocking of the canonical Wnt pathway appears promising in preclinical models. However, the impacts of potential side effects are currently unclear, as this pathway plays an important role in several physiological processes and immune-mediated inflammatory diseases. Recent advances in the understanding of Wnt/β-catenin signaling in DCs and other immune cells present promising new therapeutic opportunities for targeted regulation of this pathway to overcome immune evasion by tumors and to augment anti-tumor immunity. In this context, targeted delivery of Wnt modulators specifically to DCs and/or tumor cells using nanoparticles and antibody drug conjugates represents a promising approach for the development of novel combinatorial anti-cancer immunotherapies. This will aid in overcoming the toxicity and potential side effects associated with Wnt inhibitors.

## Summary

The central role of the Wnt pathway in regulating diverse biological processes has been appreciated for a long time. Even immunologists have recognized for decades that aberrant Wnt signaling occurs in several tumors. However, it is only recently that immunologists have begun to explore the cellular and molecular mechanisms by which the Wnt signaling pathway exerts its effects on the innate and adaptive immune systems. As evident from the discussion above, several recent observations have highlighted novel mechanisms by which the canonical Wnt signaling cascade in DCs regulates immune suppression, and the same pathway in tumors is associated with the evasion of anti-tumor immunity. In addition, preclinical studies have shown that targeting the canonical Wnt signaling pathway represents a promising approach to overcome immune evasion by tumors and promote anti-tumor immunity by potently activating DCs. However, several important questions remain unexplored (Table 2). For example, how Wnt signaling shape the innate immune functions of migratory DCs vs. non-migratory DCs in the TME? How are signals from dying cells (chemotherapy), DAMPs, and endogenous TLR ligands integrated with signals from Wnts, and what is the consequence on anti-tumor immunity? What are the effects of Wnt inhibitors plus TLR vaccine adjuvants on anti-tumor immune responses? What are the consequences of blocking the Wnt/β-catenin pathway using inhibitors as possible adjuvants plus ICI on anti-tumor immune responses? Furthermore, a major unanswered question is the extent to which the tumor-specific differences in Wnt-signaling impacts the evasion of anti-tumor immunity. Clearly, discovering answers to these questions is likely to unravel molecular mechanisms by which Wnts play a pervasive and central role in regulating anti-tumor immune response. This in turn is likely to be of great value in the design of immunotherapies against a whole range of human cancers.

**Table 2:** Some knowledge gaps in understanding how Wnts regulate anti-tumor immunity.

The role of the canonical Wnt signaling in shaping the innate immune functions of DC subsets (CD103^+^/CD8a^+^ DCs vs. CD11b^+^ DCs vs. pDCs). How Wnt signaling shape the innate immune functions of migratory vs. non-migratory DCs in the TME?
Immune cell type-specific differences in Wnt/β-catenin signaling in the TME (e.g., DCs vs. TAMs vs. MDSCs vs. Tregs vs. CTLs)
The role of the canonical Wnt signaling in intercellular cooperation in the TME and their relative contributions to anti-tumor immunity induction in a variety of tumor settings
Tumor-specific differences in Wnt-signaling and its impact on anti-tumor immunity (e.g., Melanoma vs. intestinal cancer vs. liver cancer vs. breast cancer)
The role of the non-canonical Wnt pathways in regulating the induction and maintenance anti-tumor immunity
How are signals from dying cells (chemotherapy), DAMPs, and endogenous TLR ligands integrated with signals from Wnts, and their effect on anti-tumor immunity? Consequence of Wnt inhibitors plus TLR vaccine adjuvants on anti-tumor immune responses
The role of Wnts in regulating anti-tumor immunity to immune checkpoint inhibitors (ICI), such as anti-PD1, anti-CTLA4, or anti-PD-L1. Consequence of blocking the Wnt/β-catenin (inhibitors as possible adjuvants) plus ICI on anti-tumor immune responses

## Author Contributions

AS, MH, PP, and SM have performed bibliographic researches. AS, PP, and SM have drafted the manuscript.

### Conflict of Interest

The authors declare that the research was conducted in the absence of any commercial or financial relationships that could be construed as a potential conflict of interest.
